# The latent structure of Acute Stress Disorder symptoms in trauma‐exposed children and adolescents

**DOI:** 10.1111/jcpp.12597

**Published:** 2016-07-30

**Authors:** Anna McKinnon, Richard Meiser‐Stedman, Peter Watson, Clare Dixon, Nancy Kassam‐Adams, Anke Ehlers, Flaura Winston, Patrick Smith, William Yule, Tim Dalgleish

**Affiliations:** ^1^ Centre for Emotional HealthMacquarie UniversitySydneyNSWAustralia; ^2^ Department of Clinical PsychologyNorwich Medical SchoolUniversity of East AngliaNorwichUK; ^3^ Medical Research Council Cognition and Brain Sciences UnitCambridgeUK; ^4^ Department of PsychologyUniversity of BathSomersetUK; ^5^ Center for Injury Research and PreventionThe Children's Hospital of PhiladelphiaPhiladelphiaPAUSA; ^6^ Department of Experimental PsychologyUniversity of OxfordOxfordUK; ^7^ Institute of Psychiatry, Psychology, and NeuroscienceKings College LondonLondonUK

**Keywords:** Acute Stress Disorder, DSM‐5, factor analysis, children, post‐traumatic stress disorder

## Abstract

**Background:**

The revision of Acute Stress Disorder (ASD) in the DSM‐5 (*DSM‐5*, 2013) proposes a cluster‐free model of ASD symptoms in both adults and youth. Published evaluations of competing models of ASD clustering in youth have rarely been examined.

**Methods:**

We used Confirmatory Factor Analysis (combined with multigroup invariance tests) to explore the latent structure of ASD symptoms in a trauma‐exposed sample of children and young people (*N* = 594). The DSM‐5 structure was compared with the previous DSM‐IV conceptualization (4‐factor), and two alternative models proposed in the literature (3‐factor; 5‐factor). Model fit was examined using goodness‐of‐fit indices. We also established DSM‐5 ASD prevalence rates relative to DSM‐IV ASD, and the ability of these models to classify children impaired by their symptoms.

**Results:**

Based on both the Bayesian Information Criterion, the interfactor correlations and invariance testing, the 3‐factor model best accounted for the profile of ASD symptoms. DSM‐5 ASD led to slightly higher prevalence rates than DSM‐IV ASD and performed similarly to DSM‐IV with respect to categorising children impaired by their symptoms. Modifying the DSM‐5 ASD algorithm to a 3+ or 4+ symptom requirement was the strongest predictor of impairment.

**Conclusions:**

These findings suggest that a uni‐factorial general‐distress model is not the optimal model of capturing the latent structure of ASD symptom profiles in youth and that modifying the current DSM‐5 9+ symptom algorithm could potentially lead to a more developmentally sensitive conceptualization.

## Introduction

While it is common for children to display stress responses in the first few weeks following a trauma, only a minority will go on to develop Posttraumatic Stress Disorder (PTSD; Bryant, Mayou, Wiggs, Ehlers, & Stores, 2004; Dalgleish et al., 2008; Kassam‐Adams & Winston, 2004; Meiser‐Stedman, Smith, Glucksman, Yule, & Dalgleish, 2008). This has prompted debate about the clinical utility of classifying these early post‐trauma responses using an Acute Stress Disorder (ASD) diagnosis (Bryant, 2011). First introduced in the DSM‐IV (American Psychiatric Association (APA), 1994) to identify individuals likely to develop subsequent PTSD (only diagnosable 1‐month post‐trauma), symptoms of ASD were arranged into re‐experiencing, avoidance, and arousal clusters, mirroring the structure of DSM‐IV PTSD closely. DSM‐IV ASD also included a dissociation cluster, based on research indicating that dissociation was a prognostic indicator of later pathology (Bryant 2011). This DSM‐IV conceptualization operationalizes the idea that the distinctive symptom clusters of later PTSD actually develop within the first 2 weeks of a trauma, and, in addition, that dissociation is a distinctive feature of those that will later develop chronic PTSD. However, this ASD model has poor predictive validity, and the overly restrictive nature of the dissociation criterion means that adults and children can miss out on an ASD diagnosis despite having clinically significant problems in other symptom domains (Harvey & Bryant, 1998; Meiser‐Stedman, Yule, Smith, Glucksman, & Dalgleish, 2005). Six confirmatory factor analytic tests (Armour, Shevlin, & Elkit, 2013; Brooks et al., 2008; Hansen, Armour, & Elklit, 2012; Hansen, Lasgaard, & Elklit, 2013; Wang, Li, Shi, Zhang, & Shen, 2010) carried out in adults have evaluated the DSM‐IV structure (see Table 1) of ASD compared to alternative conceptualizations derived from the PTSD literature (with the addition of a dissociation criterion), showing it is the best fitting model in only two of these six studies (Brooks et al., 2008; Hansen et al., 2013; Refer to Table S1, for a summary of study findings).

**Table 1:** Model specifications for alternative factor models of ASD

DSM‐5 ASD symptoms	Model 1 DSM‐5 1‐Factor	Model 2 Alternate 3‐Factor	Model 3 DSM‐IV 4‐Factor^a^	Model 4 Alternate 5‐Factor
B1. Intrusive memories, thoughts	ASD	Re‐ex/Ar	Re‐ex	Re‐ex
B2. Nightmares	ASD	Re‐ex/Ar	Re‐ex	Re‐ex
B3. Flashbacks	ASD	Re‐ex/Ar	Re‐ex	Re‐ex
B4a. Psychological distress to reminders^a^	ASD	Re‐ex/Ar	Re‐ex	Re‐ex
B4b. Physiological reactivity^a^	ASD	Re‐ex/Ar	Re‐ex	Re‐ex
B5. Emotional numbing^b^	ASD	Diss	Diss	Diss
B6a. Altered sense of reality: loss of awareness^c^	ASD	Diss	Diss	Diss
B6b. Altered sense of reality: Derealization^c^	ASD	Diss	Diss	Diss
B6c. Altered sense of reality: Depersonalization^c^	ASD	Diss	Diss	Diss
B7. Amnesia	ASD	Diss	Diss	Diss
B8. Avoid thinking/conversations	ASD	Av	Av	Av
B9. Avoid places/things/people	ASD	Av	Av	Av
B10. Difficulty sleeping	ASD	Re‐ex/Ar	Ar	Dys‐Ar
B11. Irritability	ASD	Re‐ex/Ar	Ar	Dys‐Ar
B12 Difficulty concentrating	ASD	Re‐ex/Ar	Ar	Dys‐Ar
B13. Hyper‐vigilance	ASD	Re‐ex/Ar	Ar	Anx‐Ar
B14. Startle	ASD	Re‐ex/Ar	Ar	Anx‐ar

Diss, dissociation; Av, avoidance; Ar, arousal; Re‐ex, re‐experiencing; Dys‐Ar, dysphoric arousal; Anx‐ar, anxious arousal; ASD, Acute Stress Disorder.

a DSM‐IV symptoms (C: psychological distress to reminders; E: Physiological reactivity) are grouped under the one symptom in DSM‐5 (DSM‐5 B4).

b DSM‐IV emotional numbing (B1) is reworded in DSM‐5 to describe absence of positive emotional experiences (DSM‐5 B5).

c DSM‐IV reduction in awareness of surroundings (B2), derealization (B3), and depersonalization (B4) are grouped under DSM‐5 Altered Sense of Reality (B)6.

Based on these sets of findings, there was a radical overhaul of ASD in the DSM‐5 (APA, 2013). Along with minor symptom changes and the revision of Criterion A describing the traumatic event, the DSM‐IV requirement of at least one symptom from a set of distinct symptom clusters was removed (although five distinct clusters were retained as an organizing principal for the different symptoms). This addressed the potentially unhelpful requirement for dissociation to be mandatory for a positive diagnosis. Instead, for a diagnosis of ASD, the DSM‐5 specifies experiencing nine symptoms from a single list of 14 (down from the DSM‐IV list of 17 symptoms) to be present. The DSM‐5 ASD thus posits a unifactorial ‘general‐distress model’. The rationale behind this change was that no compelling support for any given model of the ASD symptom structure had clearly emerged from factor analytic investigations and consequently the structure of early traumatic stress responses was considered to be too variable for distinct and reliable clustering patterns to occur (Bryant 2011). The advantage of a ‘general‐distress’ model, it was proposed, is the potential to better identify individuals at risk of developing a range of psychological disorders in the future, not just PTSD (Bryant 2011).

The removal of diagnostic clustering in ASD was a bold move. There are significant merits to elucidating a valid and reliable ASD clustering system. First, there are concerns that ASD does not represent a distinct disorder from PTSD (Brewin, Andrews, & Rose, 2003). Examining the validity of putative ASD symptom clusters, in particular the unique dissociation symptoms, is critical to the case for ASD as a distinct disorder. This argument extends beyond ASD and PTSD. Many of the symptoms of both disorders overlap with other mood and anxiety syndromes calling some to question the validity of stress disorders as a separable diagnostic entity (Brady, Killeen, Brewerton, & Lucerini, 2000). Evaluating the validity of symptom clusters unique to the stress disorders (notably the re‐experiencing symptoms) is critical in establishing the validity of this class of disorder. Secondly, elucidating potential symptom clusters can improve our understanding of the aetiology and maintenance of ASD and of later PTSD, by identifying risk factors for specific clusters/factors of symptoms, and revealing relationships between ASD factors and ongoing impairment, including later PTSD. Third, there may be underlying cognitive or biological mechanisms that only relate to a particular group of symptoms, and clustering allows these to be better identified (Frances & Widiger, 2012). Such relationships might not be detectable if clustering were removed, making it difficult to refine the specific pathways necessary for the development of treatment models that target these underlying processes. Fourth, clusters make it easier for new theoretical approaches such as the Research Domains Criteria (RDoC; Insel et al., 2010) and other trans‐diagnostic approaches to evaluate whether the psychological constructs/outcomes (e.g. intrusive memories) they aim to evaluate are consistent or different across disparate disorders. Finally, a cluster‐free diagnostic algorithm has potential implications for the prevalence rates of the disorder, relative to a cluster‐based algorithm (Magruder & Calderone, 2000; Mullins‐Sweatt, Lengel, & DeShong, 2016), with important implications for the provision of clinical care. It may be that particular clusters have a stronger relationship with clinical impairment and a cluster‐free approach impedes greater understanding of such potential relationships.

The body of empirical, modelling, and theoretical work around the diagnosis of ASD has to date focused almost entirely on the conceptualization of ASD in adults with little corresponding analysis of the diagnosis in youth. Reflecting this, the DSM‐5 draws no distinctions across the age range in terms of ASD. However, there are several putative issues concerning the application of the DSM‐5 ASD model to youth. Firstly, the model contains no developmental adaptations in contrast to the DSM‐5 conceptualization of PTSD and to a growing body of work suggesting that stress disorders present differently in adults relative to not just young children but also older children and adolescents (Meiser‐Stedman et al., 2008). In support of this, research indicates the tendency to express individual PTSD symptoms is impacted by age (Chen, Lin, Tseng, & Wu, 2002). Secondly, question marks have been raised about whether the ≥9 symptom requirement would have greater clinical utility by reducing the requirement to 3–4 symptoms (Kassam‐Adams et al., 2012). Thirdly, unlike in adults, there has been almost no examination of the factor structure of ASD symptoms in youth with the exception of a study of older Filipino adolescents exposed to a flash flood, which supported the 5‐factor dysphoria model (Mordeno & Cue, 2015). Taken together, the case against a clustered approach is less clear for traumatized youth and a proper evaluation of symptom clustering immediately post‐trauma in younger populations is required.

Given this, the study had four inter‐related aims. The first was to carry out a CFA of ASD symptoms in youth comparing the unifactorial, cluster‐free DSM‐5 approach with other models derived from the adult field (Table 1). To do this, we carried out a CFA of the 17 DSM‐IV ASD symptoms (these include the 14 DSM‐5 symptoms but allow us to also test the DSM‐IV model) in youth aged 6–17 years, who had experienced a discrete one‐off trauma, using pooled data from four sites. Data‐pooling is advantageous over single site CFA's and standard review approaches based on mean data (e.g. meta‐analysis, systematic reviews) as robust statistical techniques can be used to assess the potential influence of moderators (i.e. recruitment site, trauma type) of factor structure. Secondly, we compared ASD prevalence rates in our sample for the current DSM‐5 diagnosis relative to the previous DSM‐IV diagnosis. In youth, between 5% and 25% of children and young people suffer from ASD according to DSM‐IV (Bryant et al., 2004; Dalgleish et al., 2008; Kassam‐Adams & Winston, 2004; Meiser‐Stedman et al., 2008). Preliminary examinations of the DSM‐5 algorithm in youth suggests that the new DSM‐5 model would lead to substantially lower prevalence in children (Kassam‐Adams et al., 2012), but this finding must be replicated. Thirdly, we investigated whether the unifactorial ‘general‐distress’ model was a better predictor of clinical impairment relative to the DSM‐IV model. Finally, following from the work of Kassam‐Adams et al. (2012), we investigated whether the DSM‐5 requirement of 9+ symptoms was the best predictor of impairment relative to lower symptom thresholds.

## Method

### Sample

Data for 594 children and young people who had been exposed to a Criterion A discrete stressor within the previous 4 weeks (Mean age = 12.55 years, *SD* = 2.99, Range = 6–17 years) were pooled from centres in East Anglia (EA; *n* = 189, 8–16 years), Oxford (*n* = 65, 6–17 years), London (Sample 1: *n* = 59, 7–10 years; Sample 2: *n* = 92, 10–16 years) and Philadelphia (*n* = 189, 6–17 years). Children were recruited from either an inpatient setting or emergency department. Each study received ethics approval from the local recruitment site. Informed consent was obtained from adult carers/parents and assent from young people.

With the exception of EA, details of recruitment flow are published elsewhere (Bryant et al., 2004; Dalgleish et al., 2008; Kassam‐Adams & Winston, 2004; Meiser‐Stedman et al., 2008). In EA, the inclusion criteria were consistent with the other centres as follows: any event that involved the threat of death, severe injury, or threat to bodily integrity, or witnessing such an event [typically road traffic collisions (RTCs) and assaults]. The exclusion criteria were: intellectual disability; assaults by the young person's caregiver or close relative; being unconscious for >15 min; not being fluent in English; ongoing exposure to threat; history of organic brain damage; and significant risk of self‐harm. A member of the clinical care team at the hospital identified cases from medical records and invited families to participate by letter (opt‐out consent).

The characteristics of the final sample are presented in Table 2. The Oxford and Philadelphia samples recruited children and young people involved in RTC's. The EA sample included victims of assaults,^1^ RTC's and accidental injuries and the London sample comprised assault and RTC victims. The majority of the final sample had experienced RTC's (*n* = 441), followed by assaults (*n* = 87) and then accidental injuries (*n* = 66). The proportions of children endorsing at least one symptom from individual clusters ranged from 52% for the avoidance cluster to 76% for the dissociation cluster.

**Table 2:** Demographic characteristics and trauma‐related characteristics for the four sites

	Descriptives				Total
	East Anglia	London	Oxford	Philadelphia	
Demographics					
*n*, %	32 (*n *=* *189)	25 (*n *=* *151)	11 (*n *=* *65)	32 (*n *=* *189)	100 (*n *=* *594)
Age, *M* (*SD*)	14.17 (2.89)	11.94 (3.00)	12.67 (2.68)	11.39 (2.41)	12.56 (2.99)
6–8 years, %	5.8 (*n* = 11)	19 (*n* = 28)	11 (*n* = 7)	14 (*n* = 27)	12 (*n* = 73)
9–12 years, %	30 (*n* = 56)	41 (*n* = 62)	42 (*n* = 27)	55 (*n* = 103)	42 (*n* = 248)
13–16 years, %	65 (*n* = 122)	40 (*n* = 61)	48 (*n* = 31)	31 (*n* = 59)	46 (*n* = 273)
Male gender, %	56 (*n *=* *107)	58 (*n *=* *88)	58 (*n *=* *38)	76 (*n *=* *144)	64 (*n *=* *377)
Trauma type, %					
Assault	19 (*n *=* *35)	34 (*n *=* *52)	0 (*n* = 0)	0 (*n* = 0)	15 (*n *=* *87)
Road traffic collision	47 (*n *=* *88)	66 (*n *=* *99)	100 (*n *=* *65)	100 (*n *=* *189)	74 (*n *=* *441)
Accidental injury, %	35 (*n *=* *66)	0 (*n* = 0)	0 (*n* = 0)	0 (*n* = 0)	11 (*n *=* *66)
Full DSM‐5 ASD met, %	13 (*n *=* *25)	23 (*n *=* *35)	28 (*n *=* *12)	5 (*n *=* *9)	14 (*n *=* *103)

ASD, Acute Stress Disorder.

### Measures

Consistent with previous approaches (Meiser‐Stedman et al., 2008), we pooled data across different DSM‐IV ASD instruments. DSM‐IV ASD symptoms were indexed using well‐validated measures administered 2–4 weeks post‐trauma.^2^ Measures (described for each site below) were obtained via home interviews except at the EA site where measures were obtained over the phone.

#### Philadelphia

The Children's Acute Stress Questionnaire (CASQ; Kassam‐Adams, 2006) is a 25‐item self‐report instrument with good internal consistency (*α* = .86), test‐retest reliability (*r *=* *.76 for the total subscale and *r *=* *.59–.68 for individual subscales), and convergent validity (*r *=* *.77 with the Child and Adolescent Trauma Symptom Scale; March, 1999).

All UK sites used structured clinical interviews to assess symptoms. As there are no validated interviews of ASD in children and young people, researchers added developmentally appropriate dissociation items derived from the adult ASD literature to existing PTSD interviews, consistent with previous studies (Bryant et al., 2004; Dalgleish et al., 2008; Kassam‐Adams & Winston, 2004; Meiser‐Stedman et al., 2008).

#### East Anglia

The Children's Posttraumatic Stress Disorders Inventory (CPTSD‐I; Saigh et al., 2000) is a structured interview for assessing PTSD in children and adolescents that has excellent internal consistency, test‐retest reliability, and inter‐rater reliability (Saigh et al., 2000; Yasik et al., 2001).

#### London

Sample 1 (*n *=* *92 cases) completed The Anxiety Disorders Interview Schedule – Child Version (ADIS‐C; Silverman & Albano, 1996). The ADIS‐C contains 27 items measuring symptoms of PTSD in addition to a single item assessing impairment. Sample 2 (*n *=* *59) completed the Clinician Administered PTSD Scale – Child and Adolescent Version (CAPS‐CA; Nader et al., 1998), which indexes both the frequency and intensity of PTSD symptoms.

#### Oxford

A combination of structured clinical interview and self‐report measures determined ASD diagnosis. The widely established Children's Impact of Event Scale (IES‐8; Dyregrov & Yule, 1995), an 8‐item self‐report measure of intrusion and avoidance symptoms, was used to assess symptoms on a 4‐point scale (Not at all = 0; Rarely = 1; Sometimes = 3; Often = 5). The Child Post‐Traumatic Stress Research Index (CPTSD‐RI; Pynoos et al., 1987), a systematic clinical assessment of PTSD with widely established psychometric properties, was administered to cover items not contained in the IES‐8 (i.e. arousal items).

#### Impairment ratings

Positive categorical impairment ratings were calculated according to whether the young person endorsed problems in at least one ASD Criterion F area of functioning (e.g. school, family, and social) on each of the instruments described above.

### Data analysis

#### Confirmatory factor analysis

Four CFA models were specified (Table 1). Due to the dichotomous nature of the items, tetrachoric inter‐item correlations were estimated, and covariate adjustment then made within EQS v6.1 software (Bentler, 2006). A preliminary factor analysis using direct oblimin rotation was carried out to obtain an estimate of the size of the factor loadings for marker variables in need of scaling in the subsequent analysis (Loehlin, 1992). Mardia's multivariate kurtosis coefficient as recommended by, for example, Raykov and Marcoulides (2006) and its normalized estimate were used to assess whether the multivariate distribution of all the observed variables deviated from normality. This statistic, and the univariate item skew of >1.5, suggested non‐normality in the items. Robust maximum likelihood estimation was therefore used to fit the factor models in this sample of intermediate size (Lee, Poon, & Bentler, 1995). These estimates also perform better than uncorrected statistics where the normal distribution assumption is false and better than a distribution‐free method in all but the largest samples (Chou, Bentler, & Satorra, 1991; Hu, Bentler, & Kano, 1992). The tetrachoric correlation is recommended as a measure of association between pairs of categorical variables (Tabachnick & Fidell, 2007). The Satorra‐Bentler scaled *χ* ^2^ goodness of fit test (Satorra & Bentler, 1994) was used to index the goodness of fit for each model. The test is sensitive to large sample sizes (Tabachnick & Fidell, 2007) and a cut‐off of a *χ* ^2^: *df* of ≤3 indicates an acceptable fit.

Goodness‐of‐fit indices as recommended by Bentler (2007) were used: the Comparative Fit Index (CFI); Tucker‐Lewis Index (TLI); and the Root Mean Square Error of Approximation (RMSEA) with its 90% confidence interval (Moschopoulos & Canada, 1984). Better fitting models are denoted by higher CFI and TLI, with .90 representing a good fit, and .95 an excellent fit (Kline, 2005). Better fitting models are indicated by lower RMSEA scores. RMSEA values of ≤.05, and 90% confidence intervals whose lower bound contains, or is very close to, 0, and whose upper bound is ≤.08, are thought to indicate a close fit, .05–.08 a fair fit, and .08–.10 a marginal fit by one standard deviation (Browne & Cudeck, 1992). Multiple fit indices assessed model fit as fit indices are heavily influenced by sample size, model parameters, and data‐normality (Bentler, 2007). A good model would meet at least two fit criteria, and meeting three fit criteria would only be considered necessary according to a stringent criterion (Schermelleh‐Engel, Moosbrugger, & Müller, 2003). Schwarz's Bayesian information criterion model (BIC) combines goodness of fit with the number of model parameters needed to allow for model comparisons. BIC scores that are ≥10 points lower than the next lowest model are evidence for the superiority of one model (Raftery, 1995). Factor loadings of ≥.30 are needed for an item to be considered of practical significance (Hair, Black, Babin, Anderson, & Tatham, 2006).

MIMIC modelling was used to explore the moderating effects of recruitment site (Brown, 2006). A series of dummy variables was created, which were then specified in the structural equation for each of the items. To determine whether interpersonal (i.e. assault; *n* = 87 cases) and noninterpersonal (i.e. RTC and accidental injuries; *n* = 507) experiences led to a distinct profile of PTSD symptoms (cf. Shevlin & Elklit, 2011), a multiple‐sample group analysis (Bentler, 2006) was carried out in which tetrachoric correlation matrices were specified. In each analysis, factors that held univariate correlations to any ASD items were also covaried; in this case age and sub‐ASD (whether individuals met all of the DSM‐IV ASD criteria except dissociation – a useful index of clinical status given the problems with the dissociation criterion^3^) were removed by specifying them in the structural equation for each item.

## Results

### The latent structure of ASD in children and young people

Table 3 presents the fit indices for the four models for the full sample. The *χ* ^2^ statistics for each model were significant, and all but the 1‐factor DSM‐5 model met the pre‐requisite of a *χ* ^2^: *df* of ≤ 3. The 1‐factor DSM‐5 model was a good fit to the data (≥ .90) according to the CFI but not according to the TLI. The 3‐, 4‐ (DSM‐IV), and 5‐factor models were all an excellent fit to the data according to both fit indices (CFI and TLI ≥ .95). According to RMSEA scores, the 1‐factor DSM‐5 model was a fair fit (i.e. RMSEA ≤ .08), whereas the 3‐ 4‐ and 5‐factor models were all a close fit of the data (RMSEA ≤ .05). The lower bound confidence intervals within the RMSEA were also close to 0 for the three more complex models, indicating a higher level of precision. The model BIC highlighted the 3‐factor structure as the preferred model, in that this model was 17 points lower than the next lowest model. Inter‐factor correlations for the three factor model ranged between *r* = .50 (*p* < .001) and .80 (*p* < .001).

**Table 3:** Fit Indices for the four alternative ASD models (*N* = 594) in the pooled sample

Item models	Satorra‐Bentler *χ* ^2^(*df*)^a^	*p*	BIC	CFI	RMSEA; 90% CI	TLI
1 Factor	*χ* ^2^(118) = 391.78	<.001	−361.87	.90	.063; 0.056, 0.069	.89
**3 Factors** b	***χ*** ^**2**^ **(116) = 234.09**	**<.001**	−**506.79**	**.96**	**.041; 0.034, 0.049**	**.95**
4 Factors	*χ* ^2^(113) = 231.10	<.001	−490.61	.96	.042; 0.034, 0.050	.95
5 Factors	*χ* ^2^(109) = 207.14	<.001	−489.03	.97	.039; 0.031, 0.047	.96

ASD, Acute Stress Disorder; BIC, Bayesian Information Criterion; CFI, comparative fit index; RMSEA, root mean square error of approximation; TLI, Tucker‐Lewis Index.

a Satorra Bentler *χ* ^2^.

b Models in bold indicate the best fitting model.

We sought to determine the generalizability of this best‐supported 3‐factor model across: (a) recruitment site; and, (b) trauma type. Similar results were obtained when site differences were taken into account. The three factor model had the lowest BIC (by over 100) and an acceptable fit according to both CFI (≥ .90) and RMSEA (≤ .08) although not by TLI (= .85 < .90) or *χ* ^2^: *df* (= 3.3 > 3). The multisample CFA assessing differences in model fit across trauma type also showed the three factor model had the lowest BIC (by over 100), a *χ* ^2^: *df* of ≤ 3 and good fits using CFI (≥ .90), RMSEA (≤ .05) and TLI (≥ .90).

The factor loadings for the 3‐factor model in the full sample are presented in Table 4. The pre‐requisite of a factor loading of ≥ .30 was met for all items apart from amnesia for the trauma (DSM‐5 B7), which poorly loaded onto all other factors.^4^

**Table 4:** Standardized factor loadings (standard errors) for the ASD 3‐factor model in the pooled sample (*N* = 594)

DsM‐5 ASD symptoms	Dissociation	Re‐experiencing/Arousal	Avoidance
B1. Intrusive memories, thoughts		.83 (.04)	
B2. Nightmares		.69 (.05)	
B3. Flashbacks		.82 (.06)	
B4a. Distress to reminders^a^		.71 (.05)	
B4b. Physiological reactivity^a^		.75 (.05)	
B5. Emotional numbing^b^	.72 (.05)		
B6a. Loss of awareness^c^	.70 (.05)		
B6b. Derealization^c^	.87 (.06)		
B6c. Depersonalization^c^	.75 (.05)		
B7. Amnesia	.16 (.06)		
B8. Avoid thinking/conversations			.83 (.05)
B9. Avoid places/things/people			.85 (.05)
B10. Difficulty sleeping		.70 (.05)	
B11. Irritability		.63 (.05)	
B12 Difficulty concentrating		.73 (.05)	
B13. Hyper‐vigilance		.75 (.05)	
B14. Startle		.77 (.04)	

ASD, Acute Stress Disorder.

a DSM‐IV symptoms (C: psychological distress to reminders; E: Physiological reactivity) are grouped under DSM‐5 B4.

b DSM‐IV emotional numbing (B1) is reworded in DSM‐5 to describe absence of positive emotional experiences (DSM‐5 B5).

c DSM‐IV reduction in awareness of surroundings (B2), derealization (B3), and depersonalization (B4) are grouped under DSM‐5 B6.

### Prevalence rates

DSM‐5 ASD (9+ symptoms) led to a 0.3% increase in prevalence of ASD relative to DSM‐IV, with rates of 13.6% and 13.3% found for the two models, respectively.

### Relationship with impairment

Two hundred and nine (35.2%) young people met the impairment criterion (i.e. whether the young person endorsed problems in at least one Criterion F area of functioning). Table 5 presents sensitivity, specificity, positive predictive value, negative predictive value, for the percentage of young people correctly classified as suffering impairment using for the DSM‐IV and DSM‐5 models. Please see Table S2 for a similar table documenting the results of alternate factor models.

**Table 5:** Performance of different symptom requirements per acute stress models to predict concurrent ratings of impairment (*N* = 594)

Model	Cluster	Frequency symptom/diagnosis (%)^a^	Sensitivity	Specificity	PPV	NPV	% correctly classified	% ASD diagnosis
Model 1: DSM‐5	One‐factor: (3+)^b^	194 (32.7)	92.82	49.35	49.87	92.68	64.6	33.0
Model 1: DSM‐5	One‐factor: (4+)^c^	178 (30.0)	85.17	63.64	55.97	88.77	71.2	30.0
Model 1: DSM‐5	One‐factor: (9+)^d^	103 (17.3)	38.77	94.29	78.64	73.93	74.7	13.6
Model 3: DSM‐IV	Four factor DSM‐IV^e^	103 (17.3)	37.80	93.77	76.70	73.52	74.1	13.3

ASD, Acute Stress Disorder; NPV, negative predictive value; PPV, positive predictive value.

a The number of cases meeting the frequency requirement per symptom cluster and diagnosis (i.e. without meeting impairment).

b 3+ symptoms from the DSM‐5 list of 14 symptoms.

c 4+ symptoms from the DSM‐5 list of 14 symptoms.

d 9+ symptoms from the DSM‐5 list of 14 symptoms.

e 3+ symptoms from the dissociation cluster and 1+ symptoms from each of re‐experiencing, avoidance and arousal clusters.

The unifactorial DSM‐5 ASD model of 9+ symptoms and DSM‐IV were remarkably similar, with DSM‐5 only marginally outperforming the DSM‐IV model in terms of sensitivity and numbers of cases correctly identified, although neither was strong. Reducing the DSM‐5 symptom requirements to either 3+ or 4+ (cf. Kassam‐Adams et al., 2012) symptoms improved the sensitivity of both models by >46 percentage points. However, this improvement was off‐set by low specificity and elevated ASD prevalence rates.

## Discussion

In this study, we aimed to evaluate the latent structure of ASD symptoms in children and young people following single incident traumatic events and compare DSM‐5 and DSM‐IV prevalence rates and their ability to categorize children impaired by their symptoms. Our findings provided mixed support for the new diagnosis in children and young people. Our exploratory examination of the 3‐factor model showed that it offered the optimal account of symptom clustering. The findings showed that the DSM‐5 ‘general‐distress’ model was a good fit for the data according to two of three fit indices, whereas the 3‐ and 4‐ and 5‐factor models were excellent fits for the data according to all three fit indices. DSM‐5 and DSM‐IV models led to very similar prevalence rates and were both poor models for categorizing children's level of impairment. The sensitivity of the unifactorial model was improved by lowering the symptom threshold from 9+ (DSM‐5) to 3+/4+ symptoms (cf. Kassam‐Adams et al., 2012).

Overall, the preferred model from a structural perspective was the 3‐factor model, with re‐experiencing and arousal symptoms clustered together, but distinct from avoidance and dissociation. It is important to note that this consistent factor structure was upheld after adjusting for, age, sub‐ASD, and site differences and comparisons across trauma type. The 3‐factor model was previously shown to meet the requisite requirements of a good fitting model in three studies of ASD in adults (Edmondson, Mills, & Park, 2010; Hansen et al., 2013; Wang et al., 2010), but this model was not evaluated in the only adolescent study on this issue (Mordeno & Cue, 2015). The findings challenge the cluster‐free approach of ASD in the DSM‐5, as clustering is clearly present acutely following traumatic experiences. The good fit of adult post‐traumatic stress models on the whole suggests that early responses to trauma are perhaps not dissimilar from chronic responses in children. Theoretically, this raises important further questions. If the symptom structure of ASD is similar to PTSD, we might surmise that there is no evidence for an early ‘general‐distress’ syndrome, but rather a distinctive early post‐traumatic stress response that proceeds (without fundamental change in symptom structure) to PTSD in some cases, but remits naturally over time without intervention in others Kassam‐Adams & Winston, 2004; Meiser‐Stedman et al., 2008, 2005). However, this is speculative as a two‐factor PTSD model (i.e. avoidance, re‐experiencing+arousal) has rarely been examined in the child literature. Future CFA studies carried out in youth should test this model to determine the continuities/discontinuities in the structure of acute and chronic trauma responses in youth. In particular, future research must consider whether dissociation symptoms are an essential feature of the ASD diagnosis, whether there is any benefit if a more liberal number of symptoms are endorsed in competing models with a stronger factor structure, or whether a dissociative subtype of ASD should be introduced.

Examination of patterns of prevalence and discriminant validity were informative. Our results indicate that the adoption of the DSM‐5 model does not have a negative impact on the detection of children impaired by their symptoms relative to the other competing models (with superior patterns of clustering). Prevalence rates for both models were within the ranges suggested by previous research of ASD in youth (Bryant et al., 2004; Dalgleish et al., 2008; Kassam‐Adams & Winston, 2004; Meiser‐Stedman et al., 2008). Replicating previous research (Kassam‐Adams et al., 2012), the DSM‐5 cluster‐free model was more closely associated with clinical impairment when the developmental changes suggested by Kassam‐Adams et al. (2012) of 3+ or 4+ symptom cut‐offs were suggested, although making such a change to the DSM would need to be carefully considered as our data indicate these lower cut‐offs would likely lead to a higher rates of false positives as well as markedly increasing prevalence rates. This might then increase the provision of treatment to children whose symptoms are in fact likely to abate over time naturally, directing valuable resources away from children potentially in greater need of help (Magruder & Calderone, 2000; Mullins‐Sweatt et al., 2016). To explore this position further, prospective studies investigating the impact of factor structure on recovery trajectories and relapse rates in youth must be carried out.

Some aspects of the present methodology merit comment. The data here were collected prior to the publication of the DSM‐5 and we therefore have no pure measure of the new B5 emotional numbing symptom that refers to an absence of positive affect only (instead we used an item asking people to indicate their recent experience of positive or negative affect). In saying that, the addition of this new symptom would not have influenced the results of the CFA analyses that suggest that cluster‐free approaches are the weakest of each of the models. We also pooled data across four sites that use different instruments on the basis that DSM diagnoses are universal and different diagnostic instruments are designed to yield the same underlying constructs. However, the low numbers in some groups (e.g. Oxford, interpersonal‐trauma) was a limitation. Furthermore, the fit of the 3‐factor model was reduced when controlling for site, although it is important to highlight that it still met the minimum fit requirement of a ‘good’ model (Schermelleh‐Engel et al., 2003). Prevalence rates of ASD varied widely across site. These differences are likely due to a number of reasons, including differing age distributions, different countries within which the studies were conducted, different distributions of trauma types recruited, and different research teams. Power limitations preclude a satisfactory examination of these factors via subanalyses.

To our knowledge, this is the first study to show that the ‘uni‐factorial’ DSM‐5 model is not the optimal account of ASD symptom structure in youth. With the publication of DSM‐5 in 2013, and its cluster‐free approach to ASD, there is a significant need to continue to more fully explore the impact of nosological models of ASD in youth on theory and clinical practice.

**Key points**

- The latent structure of Acute Stress Disorder (ASD) symptoms has never before been investigated in a sample of youth following emergency department attendance or hospital admission.
- The results of this Confirmatory Factor Analysis (CFA) investigation showed that a ‘cluster‐free’ model of ASD, the model adopted for the DSM‐5 (i.e. a ‘general‐distress’ conceptualization of early responses to trauma), was not supported in youth; instead a 3‐factor model comprising dissociation, re‐experiencing/arousal, and avoidance dimensions was found to be the preferred model.
- This study shows that the pattern of symptom clustering directly opposes the ‘general‐distress’ model of ASD in DSM‐5 and supports a continuity between the structure of acute stress reactions and chronic post‐traumatic stress (as specified in the Posttraumatic Stress Disorder diagnosis).
- The proportion of children that will develop DSM‐5 ASD following exposure to single incident trauma is approximately 13.6%.
- Reducing the DSM‐5 ASD algorithm to either three or four symptoms improved the sensitivity of DSM‐5 ASD to detect children impaired by their symptoms.

## Supporting information


**Table S1.** Confirmatory Factor Analytic studies examining the structure of Acute Stress Disorder symptoms in adults.
**Table S2.** Performance of different symptom requirements per 3‐ and 5‐factor models to predict concurrent ratings of impairment (*N* = 594).

## Notes

1 The traumas that precipitated the development of ASD needed to be one‐off in nature, for example, one‐off assaults as opposed to repeated domestic violence or physical abuse. This was either determined via the initial screening carried out with parents in the hospital or via the examination of medical records.

2 Data collected across all four sites captured the full range of DSM‐IV and DSM‐5 ASD symptoms. Changes to ASD in DSM‐5 are as follows: DSM‐IV B1 ‘emotional numbing’ has been refined to describe absence of positive emotional experiences only in DSM‐5 (B5); DSM‐IV B2 ‘loss of awareness’, B3 ‘depersonalization’ and B4 ‘derealization’ are grouped under DSM‐5 B6 ‘altered sense of reality’, and; DSM‐IV symptoms (C) psychological distress to reminders and (E) physiological reactivity are grouped under DSM‐5 B4.

3 Prevalence of Sub‐ASD varied between 10% and 49% across groups.

4 This pattern of results for factor loadings was also replicated in group invariance tests addressing recruitment site and trauma type.
