# Supplementary material for: The latent structure of Acute Stress Disorder symptoms in trauma‐exposed children and adolescents
Source: J Child Psychol Psychiatry. 2016 Jul 30;57(11):1308–16. doi: 10.1111/jcpp.12597 (PMC5091623; doi:10.1111/jcpp.12597)
Supplement: Supplementary file 1 — Table S1. Confirmatory Factor Analytic studies examining the structure of Acute Stress Disorder symptoms in adults. Table S2. Performance of different symptom requirements per 3‐ and 5‐factor models to predict concurrent ratings of impairment (N = 594). [file JCPP-57-1308-s001.docx]

## Supporting information for The latent structure of Acute Stress Disorder symptoms in trauma-exposed children and adolescents by McKinnon et al.

**Table S1**. Confirmatory Factor Analytic studies examining the structure of Acute Stress Disorder symptoms in adults.

| Study | Sample | Models tested^1^ | CFI^2^ | RMSEA^3^ |
| --- | --- | --- | --- | --- |
| Brooks et al. (2008) | *N*=587 level 1 trauma patients | ***Dissociation, re-experiencing, arousal, avoidance***  *Dissociation^1st order^, re-experiencing ^1st order^, arousal^1st order^, avoidance^1st order^, general distress^2nd^* *^order^* | .97*  .97* | .036*  .032* |
| Edmondson et al. (2010) | *N*=132 Hurricane Katrina evacuees. | Dissociation, re-experiencing, arousal, avoidance  *Dissociation,* re-experiencing*/arousal, avoidance*  *Dissociation^1s order^,* re-experiencing *^1st order^, arousal^1st order^, avoidance^1st order^, general distress^2nd order^*  ***Dissociation^1st order^ , re-experiencing ^2nd order^, avoidance^2nd order^, arousal^2nd order^*** | .86  .91*  .92*  .93* | .09  .07*  .07*  .07* |
| Wang et al. (2010) | *N*=353 Chinese earthquake victims | ***Dissociation,* re-experiencing*, arousal, avoidance***  *Dissociation, re-experiencing/arousal, avoidance*  *Dissociation, dissociative amnesia, acute posttraumatic stress reactions*  *Dissociation^1st order^,* re-experiencing *^1st order^, arousal^1st order^, avoidance ^1st order^  general distress^2nd order^* | .99*  .99*  .99*  .99* | .038*  .039*  .042*  .039* |
| Hansen et al. (2012) | *N*=404 rape and bank robbery with ASD. | ASD  Dissociation, re-experiencing /arousal, avoidance  Dissociation, re-experiencing, arousal, avoidance  **Dissociation, re-experiencing, dysphoria, arousal, avoidance** | .59  .74  .74  .81 | .085  .069*  .069*  .061* |
| Armour et al. (2013) | *N*=380 Danish rape victims | ASD  ***Dissociation, re-experiencing/arousal, avoidance***  Dissociation, re-experiencing, arousal, avoidance  Dissociation, re-experiencing, dysphoria, arousal, avoidance  Dissociation, re-experiencing, numbing, avoidance, arousal | .67  .80  .80  .81  .79 | .075*  .058*  058*  .058*  .06* |
| Hansen et al. (2013) | *N*=450 bank robbery victims. | ASD  *Dissociation^1st order^,* re-experiencing *^2nd order^, avoidance^2nd order^, arousal^2nd order^*  *Dissociation, re-experiencing /arousal, avoidance*  ***Dissociation, re-experiencing, arousal, avoidance*** | .96*  .97*  .98*  .98* | .089  .076*  .070*  .068* |

Note

^1^ Models *italicised* either had a good or excellent fit of the data according to the χ2 in addition to CFI and RMSEA fit indices. Models in **bold** are the best fitting model.

^2^Comparative Fit Index: The ratio of the difference between the χ2 for the fitted model and the null model divided by the χ2 for the null model with ≥ .90=good fit and ≥ .95=excellent fit. CFI’s meeting either criteria are marked by an asterisk.

^3^ Root Mean Square Error of approximation: The amount of unexplained variance left by the models with ≤.05 suggesting a close fit and ≤.08 suggesting a fair fit. CFI’s meeting either criteria are marked by an asterisk.

Table S2. Performance of different symptom requirements per 3- and 5-factor models to predict concurrent ratings of impairment (N=594).

| **Cluster** | **Frequency symptom/diagnosis^1^** | **Sensitivity** | **Specificity** | **PPV** | **NPV** | **% correctly classified** | **% ASD diagnosis** |
| --- | --- | --- | --- | --- | --- | --- | --- |
| 3-factor^2^ | 117 | 41.63 | 92.21 | 74.36 | 74.42 | 74.41 | 14.65% |
| 5-factor**^3^** | 69 | 33.01 | 100 | 100 | 73.3 | 76.43 | 4.88% |

*Note.* NPV=negative predictive value; PPV=positive predictive value

^1^ The number of cases meeting the frequency requirement per symptom cluster and diagnosis (i.e., without meeting impairment).

^2^ 3+ dissociation, 2+ re-experiencing/arousal, 1+ avoidance.

^3^ 3+ dissociation, 1+ re-experiencing, 1+ avoidance, 1+ arousal.
